# History of Dental Practice on the Pacific Coast

**Published:** 1863-12

**Authors:** H. H. Black

**Affiliations:** Portland, Oregon


### Correspondence.

### HISTORY OF DENTAL PRACTICE ON THE PACIFIC COAST.

EDITORS REGISTER:—I feel that an apology is due your readers for not completing my "account of the profession on the Pacific Coast" ere this; but as I shall be able to add but few items of interest, the procrastination is of little consequence.

But to Dr. Taft there is a special apology due for not complying with his kind and repeated solicitations long since. The conclusion of my last letter made brief reference to the limited encouragement extended to dental literature by the profession on the Pacific coast, which subsequent observation has shown to be attributable to the amazing ignorance of four-fifths of the practitioners whose perigrinations throughout the Pacific States and Territories is of no less notoriety than that of the Gipsies in their favorite country.

The transient condition of the masses, made so by the fabulous rise and fall of cities, together with the constant vibrations of mining excitements, seem to render the interior and mining sections of this country peculiarly adapted to the interests of the itinerant and empiric dentist. In conversation with dentists of this class, should you chance to speak of dental journals, they will assume a sarcastic smile and remark "What use has a dentist in the mines for such trash?" Speak to them of dental schools and libraries, and their reply will be "Libraries are humbugs, and will do very well for a show, but what do we want of them in a mining country where we have more use for faro-banks, ten-ounce nuggets, and revolvers."

In thus describing what is termed the "miners' dentist," *alias*, one who lives in the mining camps, supporting himself by the use of forceps and gambling tables, truth compels me to assert that many of them have heretofore occupied prominent positions in the profession, and most of them are men possessed of great daring, shrewdness and natural intellect, and who, from years of seclusion and accustomed hermitage, have learned to love a mountain life in the mining districts of the "Golden States," in preference to any spot on earth.

In addition to the class of dentists just described, the people of the "Golden Sea-board" are cursed with another class of imposters, more properly designated "tooth-carpenters," whose extreme ignorance and brazen-faced pretensions make them still more loathsome than the class first described. Of this class of dentists, but few are found in the mining camps or gambling towns, as they are wanting the moral courage to place themselves in proximity to dentists skilled in the use of pistols so peculiar to these places. But they are to be found in every town in the older and better governed portions of our country, representing themselves as being a member of some popular dental association and an "intimate and *regular* correspondent with the Dean of the faculty of the Baltimore College of Dental Surgery," and other remarks that are equally weak and sickening. Such is but a brief description of the amazing impudence of these brazen-faced pretenders, so peculiar to the localities just described.

They have taken up the profession with but a slight appreciation of its responsibilities, and an inconsiderate knowledge of its details; and have, by mal-practice and impiricism, caused much suffering, and detracted from the esteem in which it should be held. Their operations are performed for the benefit of the operator, and not for that of the patient. Their professional antecedents (pardon the term) are still less recommending,—being easily traced to an origin closely resembling that of the barber, hod-carrier, gingerbread peddler, or fish-monger.

While this description is claimed to be a correct but inadequate one of the multitude of quacks that flood the Pacific coast from Guaymas in Sonora to Victoria in British Columbia, it by no means has any reference to the honest and competent dentists to be found in San Francisco, Sacramento, Marysville, and other cities on the Pacific. We have some as good dentists as there are in the United States; but they comprise about one-fifteenth of the practitioners; and, from this, your readers can readily perceive the necessity of an ingression of a number of competent dentists to displace these miserable "tooth-carpenters." Portland would at once present an example as being in most need of this exchange.

Since the publication of my first letter on this subject, I have received letters from a great number of dentists in the Atlantic States, making inquiries as to the "society, health, and climate of this country; and also the population of the different cities, the number of dentists in each, and the comparative wealth of this country to that of the Atlantic States." In replying to these interrogations, I shall be able to give but a vague description, as it necessarily requires more time than is allotted to me. But, for the benefit of those who may think to make this country their home, I will venture a brief description: The society of the Pacific Coast is of rather a transient nature, and, except in the larger towns, is chiefly composed of men who are of every "tongue and kindred." In the older cities on the Columbia, Sacramento, American and Willamette rivers, the society is much more refined, and the numerous schools and churches furnish unmistakable proof of a refined and prosperous people. The general health of the entire Pacific Coast, from Guaymas in Sonora to New Westminster in British Columbia, surpass that of any country on the American Continent.

The climate is varied, but of singular uniformity in certain localities. The bracing winds, so much admired in San Francisco, are rarely experienced in Sacramento. The "Oregon mist" (a local name for the rainy season), seldom dampens the sun-parched streets of Los Angelos. The following table of the measurement of rains will afford your readers a better idea of the climate of Oregon:

| Months. | 1861-62. |
|---|---|
| October, | 3.98 |
| November, | 18.05 |
| December, | 98.95 |
| January, | 8.21 |
| February, | 6.02 |
| March, | 16.39 |
| Total, | 71.60 |

In the foregoing table, the snow which fell is included, and the amounts are expressed in inches and hundredths of inches.

The following table shows the number of rainy days during the twelve months ending April 31st, 1863:

| | | | |
|---|---|---|---|
| May, | 16 | November, | 3 |
| June, | 11 | December, | 20 |
| July, | 9 | January, | 24 |
| August, | 4 | February. | 21 |
| September, | 5 | March, | 13 |
| October, | 6 | April, | 13 |
| Total, | | | 145 |

The above table includes all rainy days, whether it rained all day or only a part. It also includes snowy days.

I should be pleased to furnish your readers, and particularly my dental acquaintances in Cincinnati, with a more satisfactory description of the Pacific States, and dental practice therein; but duties to practice prevent it at present. Should an opportunity offer, I may hereafter have something more descriptive to say, both of the country and the novel manner some of our "tooth-carpenters" have of "raising the wind." H. H. BLACK.

*Portland, Oregon*, Oct. 23, 1863.

